# Field Performance of Recycled Plastic Foundation for Pipeline

**DOI:** 10.3390/ma8052673

**Published:** 2015-05-19

**Authors:** Seongkyum Kim, Kwanho Lee

**Affiliations:** Department of Civil Engineering, Kongju National University, Cheonan 330-717, Korea; E-Mail: tjdrua0614@kongju.ac.kr

**Keywords:** field test, pipe deformation, recycled plastic foundation, sewer pipe

## Abstract

The incidence of failure of embedded pipelines has increased in Korea due to the increasing applied load and the improper compaction of bedding and backfill materials. To overcome these problems, a prefabricated lightweight plastic foundation using recycled plastic was developed for sewer pipelines. A small scale laboratory chamber test and two field tests were conducted to verify its construction workability and performance. From the small scale laboratory chamber test, the applied loads at 2.5% and 5.0% of deformation were 3.45 kgf/cm^2^ and 5.85 kgf/cm^2^ for Case S1, and 4.42 kgf/cm^2^ and 6.43 kgf/cm^2^ for Case S2, respectively. From the first field test, the vertical deformation of the recycled plastic foundation (Case A2) was very small. According to the analysis based on the PE pipe deformation at the connection (CN) and at the center (CT), the pipe deformation at each part for Case A1 was larger than that for Case A2, which adopted the recycled lightweight plastic foundation. From the second field test, the measured maximum settlements of Case B1 and Case B2 were 1.05 cm and 0.54 cm, respectively. The use of a plastic foundation can reduce the settlement of an embedded pipeline and be an alternative construction method.

## 1. Introduction

Modern civilization has been developed in a city-oriented manner, and infrastructure has gained increasing importance to maintain the functions of cities. Sewage pipelines are crucial city infrastructure. Pipelines are analogous to blood vessels in our body; water pipes are like the main arteries, and sewage pipelines are like veins that act as the lifeline of the city. Once sewage pipelines are constructed, they are expected to remain in use for 20 years as a highly critical national infrastructure to carry wastewater and rainfall to sewage treatment plants or reservoirs [1].

The government of the Republic of Korea declared 2002, “the first year of sewer special maintenance”, and has focused strong attention and investment on the sewage and infrastructure sectors. Because of this, the Ministry of the Environment has begun the Sewer BTL (Build-Transfer-Lease) Project and the Han River sewer improvement project, a sewer facility expansion project for upstream areas across the nation. They have also devoted more effort to ensure smooth progress of the sewer improvement project budget for the active sewer business.

**Figure 1:**
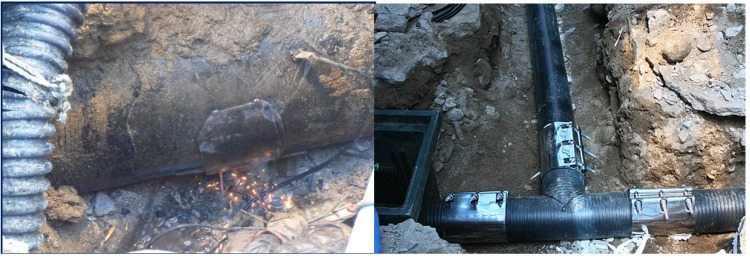
Failure of underground pipelines.

In recent years, the use of flexible pipe including thermoplastic resin pipe and thermosetting plastic resin pipe has increased. The major advantages are easy use, excellent durability, flame-retardation and easy transportation. These sewer pipes are classified as flexible plastic pipe. The performance of flexible pipe highly depends on the interaction with the surrounding soils. Korea features several types of major damage to sewage pipelines: approximately 38% are joint defects, approximately 30% are extrusions of the connection part, approximately 30% are joint connection defects, 12% are due to the accumulation of impurities inside of the pipes, 10% are cracks and 10% are other types of damage. Joint defects, joint connection defects and other defects make up approximately 70% of the total damages and are related to quality management and the degree of the compaction of backfill materials that are executed around the pipes [2,3,4]. The age of the material, sink of pipe backfill materials and various other factors affect the damage to sewage pipelines. Furthermore, pipes buried underground are not visible, and risks are not visible. Therefore, some problems may result in serious damage, as depicted in Figure 1. More severe damage can occur in a short time frame due to heavy torrential rain caused by recent climate change, inadequate drainage for rainfall, the defects in connection joints and pipe damage [5,6]. RCA is a by-product of the construction and demolition activities of concrete structures. Concrete chunks are crushed into aggregates of variable sizes depending on the field of application. Various authors have reported on the geotechnical properties of RCA in geotechnical and pavement sub-base applications [7,8]. Several researchers have stated that appropriate design methods in pipe backfilling can be set in a way that can minimize or partly prevent contaminants [9,10,11]. Among them, Rahman *et al.* [8] investigated the suitability of recycled construction and demolition materials as alternative pipe backfilling materials for storm-water and sewer pipes. Three commonly found recycled construction and demolition waste materials—crushed brick, recycled concrete aggregate and reclaimed asphalt pavement—were investigated to assess their suitability as pipe backfilling material. The physical, geotechnical and chemical properties of these construction and demolition materials were compared to specifications from the local engineering and water authorities for typical quarried materials to assess their performance as a viable substitute for virgin quarried aggregates in pipe backfilling applications.

The objective of this study was to evaluate the performance of recycled plastic foundations for sewage systems. The use of plastic foundations could reduce damage to connections of sewage pipelines and improve their safety. In this research, two different types of tests (a small chamber test and a field test) were conducted. The purpose of the small chamber test is to verify the performance of PE pipe without and with plastic foundation. This result was used to setup the test combinations or the field test. Two types of field test were conducted. The first test checked the feasibility and constructability of plastic foundations. The second test simulated the field construction to check the deflection of PE pipe, which was installed on the manhole.

## 2. Results and Discussion

### 2.1. Testing Materials

#### 2.1.1. PE Triple Wall Pipe

This study used a flexible PE triple wall pipe. The PE triple wall pipe is a flexible pipe molded by extrusion using high-density polyethylene (HDPE) [12]. This material is lightweight and easily-handled for field construction. In addition, the material is very resistant to acidic and alkaline substances. Care should be taken during construction for buoyancy and to compact the backfill materials around the pipeline [13]. Table 1 shows the standard specifications of PE pipe in Korea. In this research, the PE triple wall corrugated pipe used in this experiment featured a 250 mm inner diameter, 284 mm outer diameter, 20 mm thickness and 6000 mm length.

**Table 1:** Dimensions of polyethylene (PE) pipe in Korea.

Type	Inside (mm)	Outside (mm)	Thickness (mm)
D 150	150	180	15
D 200	200	232	16
D 250	250	284	17
D 300	300	340	20
D 350	350	398	24
D 400	400	460	30
D 450	450	510	30
D 500	500	570	35
D 600	600	694	47

#### 2.1.2. Plastic Foundation

**Figure 2:**
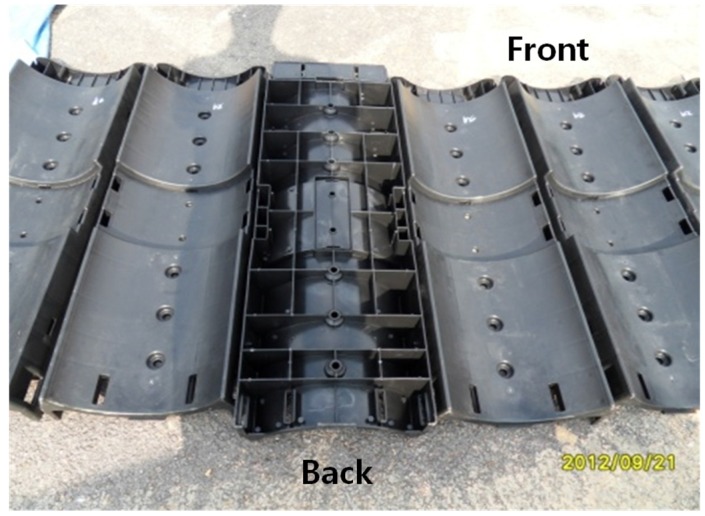
Plastic foundation by injection molding methods.

This study used a prefabricated plastic foundation with the injection molding of general plastic at high temperature and pressure, as shown in Figure 2. The injection-molded products are 1.5 m long and weighed 4 kg for ease of use. This process was simplified to meet the site conditions, and a cross section was fabricated to be applied in a straight section for testing purposes. The material used was HDPE. Injection molding products using 50% recycled plastic and 50% new plastic was applied with a 90 degree contact angle only.

The properties of the composited plastic foundation were tested to fabricate the plastic foundations for sewage pipelines. The tested properties included density and the elastic modulus according to an impact resonance test and a uniaxial compressive test [1,14,15].

The material density was 0.93 t/m^3^. The densities of the tested materials were low; specifically, this material was less dense than water. The elastic modulus was evaluated using an impact resonance testing machine. The measured elastic modulus value was 1764 N/mm^2^. The uniaxial compressive test was conducted at 18 °C (normal temperature) and −15 °C (low temperature). Its yielded values of 21.22 N/mm^2^ at room temperature and 21.22 N/mm^2^ at low temperature, which were based on a uniaxial compressive test.

#### 2.1.3. Backfill Materials

In general, natural sand is used a bedding and backfill material for sewer pipelines in Korea. The unit weight, internal friction angle and relative density were 15.46 kN/m^3^, 43.5° and 80%, respectively. The natural sand is classified as SP by ASTM D 2487. In this study, recycled *in-situ* soil from a construction site near Cheonan City, Korea, was used. The characteristics of the *in-situ* soil were relatively uniform, and its water content was approximately 14%. This soil is classified as SC by ASTM D 2487.

### 2.2. Small Scale Lab Test

#### 2.2.1. Properties of Sand Backfill

Two different testing conditions (Case S1 and Case S2) were used to verify the characteristics of deformation for buried PE pipes. The measured dry density and degree of compaction are shown in Table 2. The degrees of compaction at the top, middle and bottom were approximately 81.4%, 86.0% and 85.9%, respectively.

**Table 2:** Measured properties of sand backfill.

Items Case		Dry Density (g/cm^3^)		Degree of Compaction (%)	
		Average	Standard Deviation	Average	Standard Deviation
Case S1 (360° Sand Bedding)	Top	1.333	0.037	81.3	2.3
	Middle	1.394	0.019	85.0	1.1
	Bottom	1.408	0.031	85.8	1.9
Case S2 (50% Recycled Plastic Foundation	Top	1.336	0.013	81.5	0.8
	Middle	1.416	0.016	86.4	1.0
	Bottom	1.411	0.027	86.0	1.6

#### 2.2.2. Vertical and Lateral Deformation of PE Pipe

**Figure 3:**
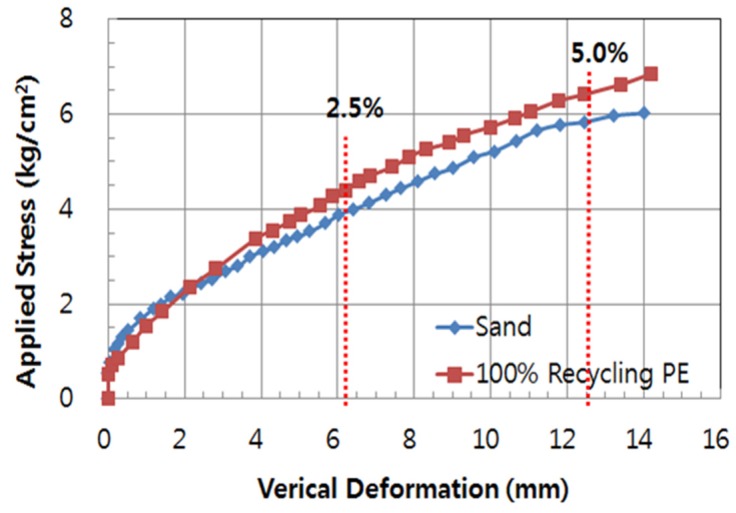
Vertical deformation of PE pipe.

Two different testing conditions (Case S1 and Case S2) were used to verify the characteristics of deformation due to the applied load that simulated traffic loading. The measured test results are shown in Table 3 and Figure 3. In Korea, the specification of allowable deformation for buried PE pipe is a 5% maximum deformation for the pipe diameter. For comparison, 2.5% and 5.0% of deformation were selected. The applied loads at 2.5% and 5.0% of deformation were 3.45 kgf/cm^2^ and 5.85 kgf/cm^2^ for Case S1, and 4.42 kgf/cm^2^ and 6.43 kgf/cm^2^ for Case S2, respectively. This means that the use of a 50% recycled plastic foundation (Case S2) can endure larger loading than that of sand bedding (Case S1).

In the case of lateral deformation, the measured deformations at 2.5% and 5% were 6.60mm and 12.51 mm for Case S1, and 5.90 mm and 11.30 mm for Case S2, respectively. Case S2 showed better resistance than Case S1.

**Table 3:** The measured vertical and lateral deformations of PE pipe.

Bedding Type		Case S1		Case S2	
Deformation Ratio According to Pipe Diameter		2.5%	5%	2.5%	5%
Vertical	Applied Stress (kgf/cm^2^)	3.45	5.85	4.42	6.43
Lateral	Deformation (mm)	6.60	12.51	5.90	11.30

#### 2.2.3. Flatness of PE Pipe

**Figure 4:**
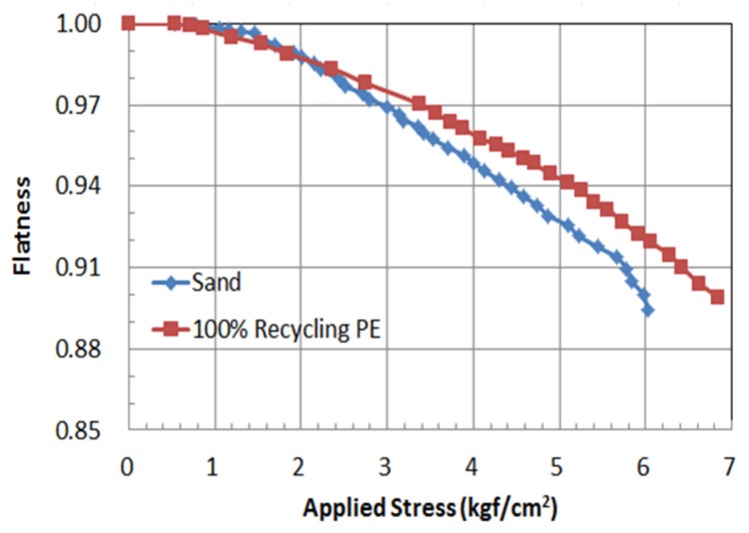
The flatness of PE pipe with applied stress.

The flatness is defined as the ratio of the vertical (compression) deformation to the lateral (tensile) deformation. A flatness 1.0 means that the pipe is a perfect circle. As the flatness number decreases below 1.0, the shape of the PE pipe is flattened. Table 4 and Figure 4 show the calculated flatness of the PE pipe for Case S1 and Case S2. In general, the calculated flatness of Case S2 is lower than that of Case S1, meaning that the PE pipe in Case S2 is more resistant than that of Case S1. The flatness of Case S1 and Case S2 at 6.0 kgf/cm^2^ of applied stress was 0.896 and 0.921, respectively. The flatness number used to calculate the flowable flux.

**Table 4:** The measured flatness of PE pipe for each case at same applied stress.

Type	Case S1			Case S2		
Applied Stress (kgf/cm^2^)	2.0	4.0	6.0	2.0	4.0	6.0
Flatness	0.988	0.949	0.896	0.988	0.960	0.921

### 2.3. First Field Test

**Figure 5:**
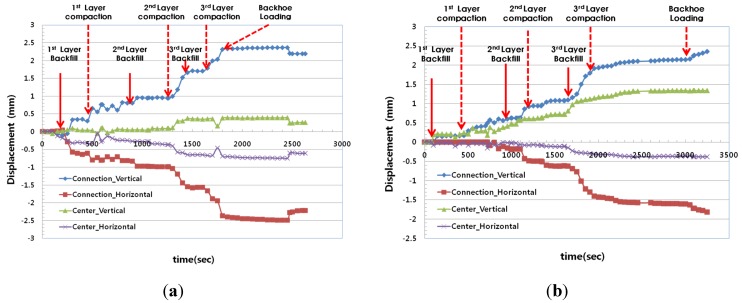
The displacement of each case with construction stages. (**a**) Case A1; (**b**) Case A2.

The vertical and horizontal deformations by LVDT at the PE pipe connections (CN) of the plastic pipe are shown in Figure 5. In general, the deformations of the plastic pipe are compressive for the vertical direction and expansive for the horizontal direction. The measured vertical deformation at the PE pipe connection (CN) of the plastic pipe was approximately 2.36 mm for Case A1 and 2.35 mm for Case A2, or 8% of the total PE pipe diameter (300 mm). The horizontal deformations at the same point are related to tensile strain. The measured deformations were 2.49 mm for Case A1, and 1.82 mm for Case A2, respectively.

The vertical and horizontal deformations of the plastic pipe at the center of the pipe (CT) were measured and are shown in Table 5, according to the construction stages. The measured horizontal deformations were 0.39 mm for Case A1, and 1.34 mm for Case A2, respectively. In Case A1, there were some deformations of the sand bedding during the compaction stages of the backfill material, which induced the deformation of the PE plastic pipe for the vertical direction. This deformation is relatively large at the connection point and small at the center of the pipe. On the other hand, the vertical deformation of the recycled plastic foundation (Case A2) is very small. This means that the vertical pressure transferred to the horizontal direction. According to the analysis based on the PE pipe deformation at the connection (CN) and at the center (CT), the pipe deformations at each part for Case A1 were larger than those for Case A2, which adopted the recycled lightweight plastic foundation. These results mean that the use of recycled lightweight plastic foundation can reduce the differential deformation of PE pipe in the longitudinal direction.

**Table 5:** Pipe Deformation at the Connection (CN) and the Center Position (CT).

Case	At Connection (CN)				At Center Position (CT)			
	Case A1		Case A2		Case A1		Case A2	
Construction Stage	Vertical	Horizontal	Vertical	Horizontal	Vertical	Horizontal	Vertical	Horizontal
After1st backfill	0.55	−0.73	0.21	−0.04	0.03	−0.05	0.29	−0.04
After 1st compaction	0.82	−0.82	0.24	−0.18	0.06	−0.24	0.41	−0.03
After 2nd backfill	0.94	−0.97	0.25	−0.02	0.04	−0.28	0.62	−0.08
After 2nd compaction	0.94	−0.99	0.39	−0.13	0.09	−0.37	0.72	−0.12
After 3rd backfill	1.18	−1.20	0.73	−0.45	0.29	−0.58	1.19	−0.32
After 3rd compaction	1.78	−1.67	1.46	−1.11	0.36	−0.67	1.30	−0.37
After backhoe loading	2.36	−2.49	2.34	−1.99	0.39	−0.75	1.34	−0.38

### 2.4. Second Field Test

**Figure 6:**
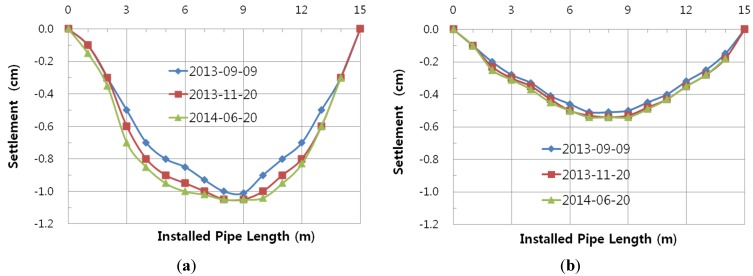
Measured settlement of Pipe. (**a**) Case B1; (**b**) Case B2.

The installed pipe length was 15 m from left to right. After installing the plastic foundation and the PE pipe, the backfill was dumped and compacted according to KS standard specifications. A dump truck with a full cargo was used as traffic loading right on the embedded pipeline. The settlements of the pipeline based on the construction level were measured using a level and are shown in Figure 6. The settlement of Case B2 with the recycled plastic foundation is smaller than that of Case B1 with the sand bedding. The measured maximum settlements of Case B1 and Case B2 are 1.05 cm and 0.54 cm, respectively. The use of a plastic foundation can reduce the settlement of the embedded pipeline.

## 3. Experimental Section

### 3.1. Small Scale Lab Test

**Figure 7:**
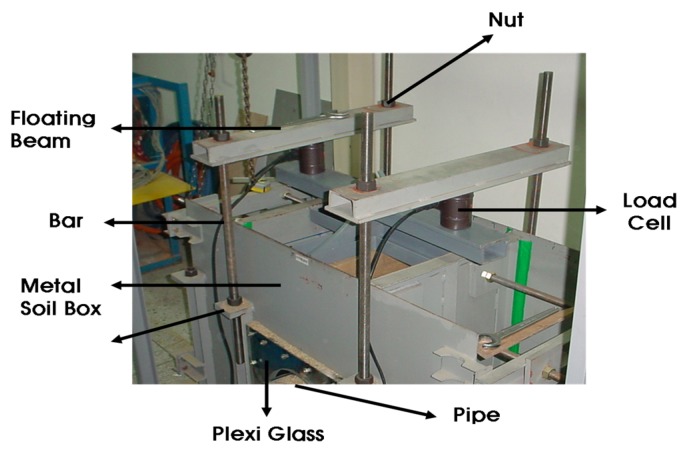
Small-scale chamber.

The small-scale chamber test was performed to evaluate the performance of the plastic foundation for pipelines. The purpose of the small chamber test was to verify the performance of the PE pipe without and with a plastic foundation. This result was used to setup the test combinations or the field test. The dimensions of the small-scale chamber were 1.4 m × 0.6 m × 0.9 m, and it was reinforced with horizontal and vertical flat metal strips, as shown in Figure 7. As observed in the figures, a square metal plate with an elliptical hole was attached to the box with nuts and bolts. A rubber membrane sheet was placed between the chambers and the plate before tightening the plate, and a 30-cm circular hole was cut in the plate to insert the pipe. The membrane was used to ensure water tightness without affecting the behavior of the pipe.

**Figure 8:**
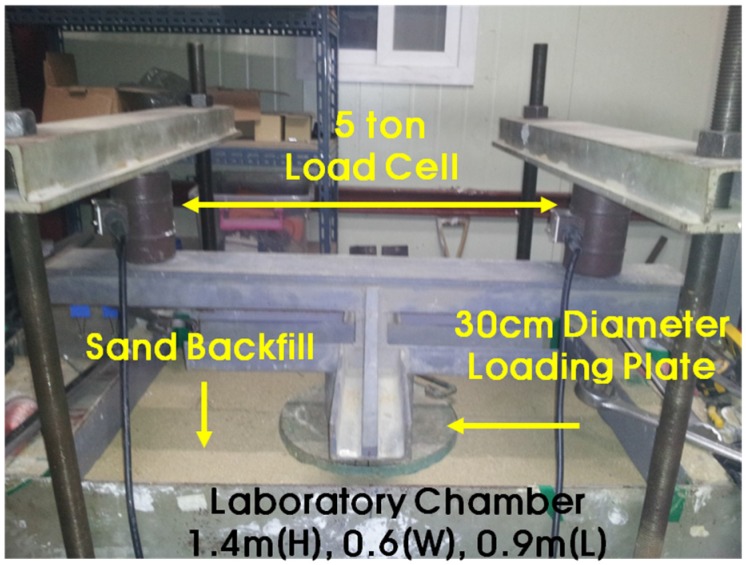
Loading system.

**Figure 9:**
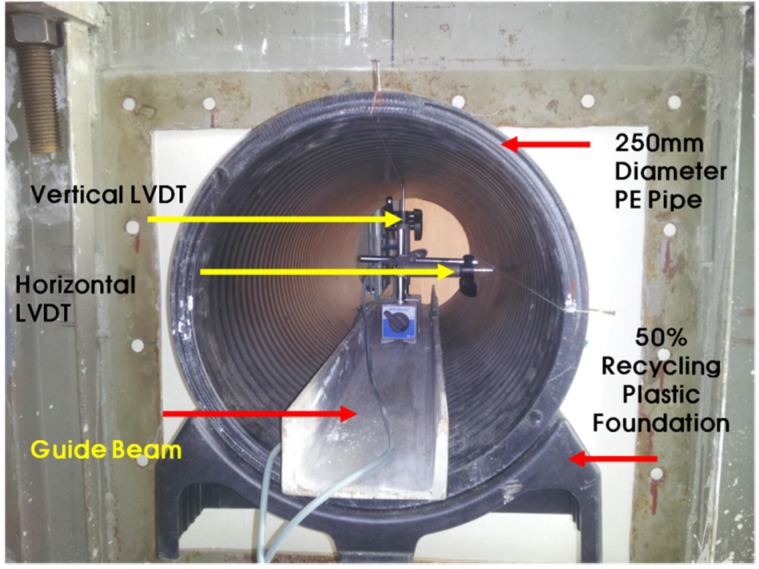
Setup of Linear Variable Differential Transducer (LVDT).

The loading system with a circular plate on top of the chamber and two metallic blocks over the plate is shown in Figure 8. A metal beam with a load cell was placed between the metal blocks and the beam. The maximum capacity of each load cell was two tons. The load cell was pre-calibrated using a 5-ton universal testing machine. A linear variable differential transformer (LVDT), shown in Figure 9, was installed to obtain the vertical and horizontal deflections of the pipe. An automated data acquisition system was used to collect the data.

**Figure 10:**
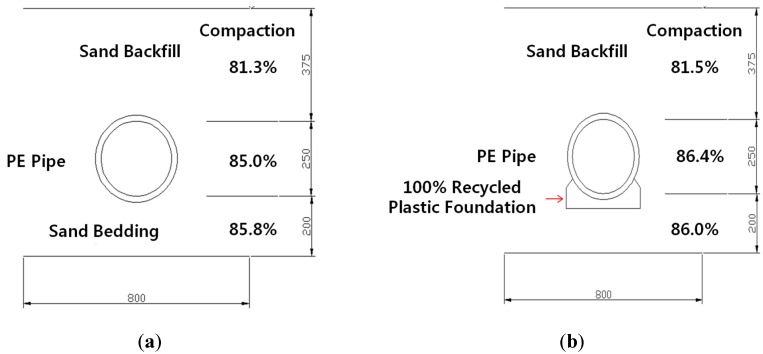
The two testing conditions, (**a**) Case S1; (**b**) Case S2.

The two different conditions of the test are presented in Table 6 and Figure 10. Natural sand and 50% recycled plastic foundation were used as a bedding material. The natural sand was used as a backfill material. A PE pipe with a 25 cm diameter and 0.7 cm thickness was installed. In the laboratory chamber test, the chamber size was limited, which could affect the test results due to boundary conditions. To minimize the boundary effects, a vinyl sheet was attached to the chamber wall to reduce the friction effect. The length of the PE pipe was 1 m. In the field, the usual length of a PE pipe is over 6 m. The short length of the PE pipe induced a smaller deflection of the PE pipe at the center of the applied load. In this project, obtaining the real deformation or deflection of PE pipe in the field was not easy. However, we obtained the general trend of deformation and deflection of the PE pipe with and without the plastic foundation.

**Table 6:** Testing cases for small scale lab test.

Type	Bedding and Foundation	Backfill
Case S1	360° Sand Bedding	Natural Sand
Case S2	50% Recycled Plastic + 50% New Plastic Foundation	Natural Sand

### 3.2. First Field Test

#### 3.2.1. Procedure of Field Construction

For the evaluation of field performance, two types of field tests with recycled plastic foundation for sewer pipelines were conducted. The first test checked the feasibility and constructability of plastic foundations. The second test simulated the field construction to check the deflection of PE pipe, which was installed on the manhole.

**Figure 11:**
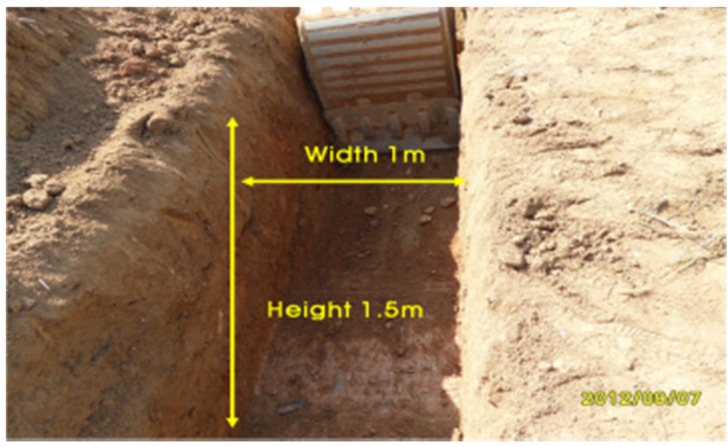
Trench excavation for the plastic foundation.

The process of field construction followed the Korean Standard Specification. As shown in Figure 11, the vertical trench was 1.5 m of deep and 1.0 m of wide.

**Figure 12:**
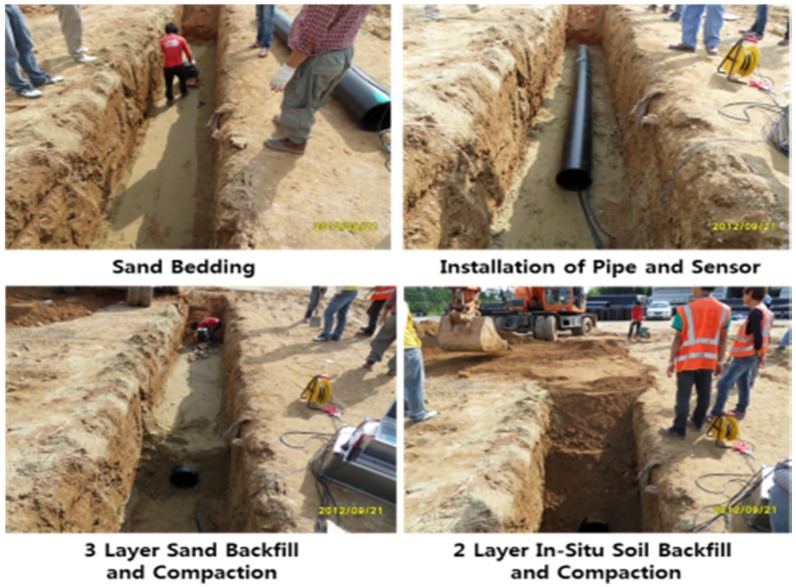
Field construction process.

Three different cases (Table 7) with different bedding materials, foundations and backfill materials were used in this research. Figure 12 shows the field construction process, including the excavation of the trench, sand bedding, pipe installation, installation of measuring instruments, backfill and compaction. The density of each compaction layer was measured, and the average relative density was over 80%.

**Table 7:** Cases for the first field test.

Type	Bedding and Foundation	Backfill
Case A1	Sand	One Layer of Sand + Two Layers of Original Soil
Case A2	50% Recycled Plastic + 50% New Plastic Foundation	Three Layers of *In-Situ* Soil

#### 3.2.2. Measuring Instruments and Load

**Figure 13:**
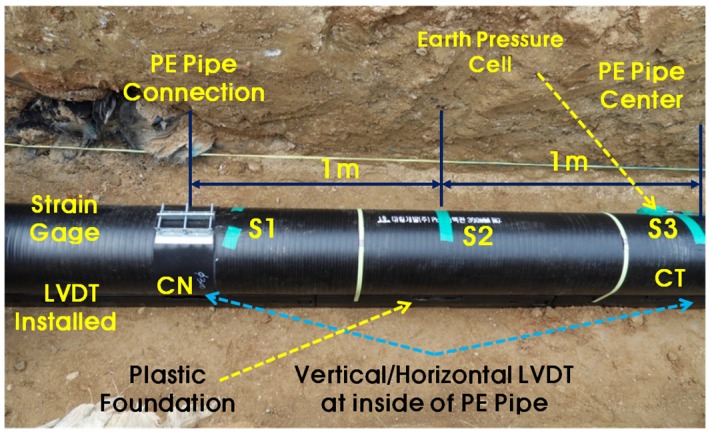
Measuring system.

The measuring instruments are shown in Figure 13. To obtain the vertical and horizontal deformations of the PE pipe, two sets of LVDT for the vertical and lateral directions were installed at two different locations, such as at the PE pipe connection (CN) and the center of the PE pipe (CT). To measure the longitudinal strain of the PE pipe, three strain gauges were installed at the PE pipe connection (S1) to the center (S3) with 1 m of intervals. The backhoe was used to simulate real traffic loads in the roadway.

### 3.3. Second Field Test

**Figure 14:**
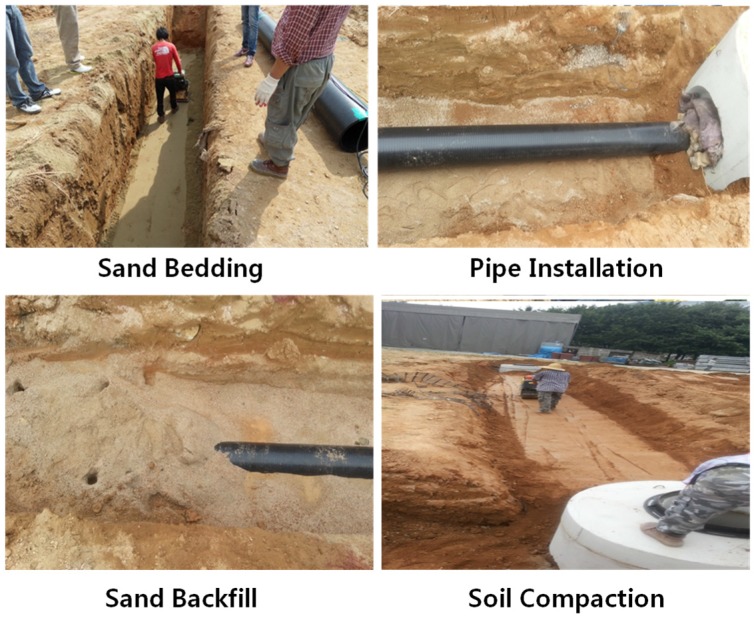
Second field construction for common process.

**Figure 15:**
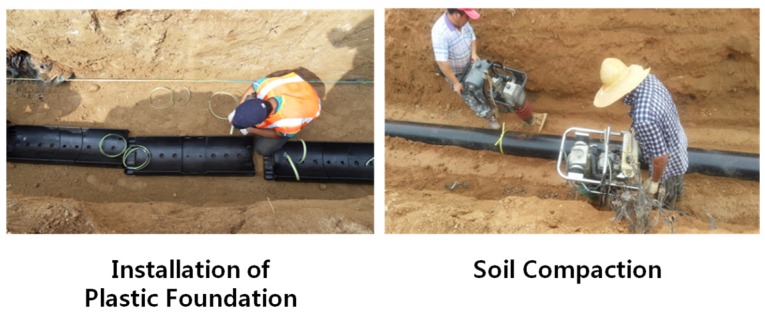
Second field construction for new process.

The second field test (Figure 14 and Figure 15) was conducted to verify the performance of the recycled lightweight plastic foundation and to evaluate the deflection of the PE pipe. The stages of field construction were very similar to the first field test. The main different was the length of the PE pipeline, which varied from 6 to 15 m, with two connection parts. The PE pipe was installed using 15 m of manhole to manhole, which means that the pipe had fixed support at the beginning and ending positions. Table 8 shows the test cases of the second field test. Case B1 is a common process for installing PE pipelines with sand bedding in Korea. Case B2 is a brand new process using the recycled lightweight plastic foundation for sewer pipelines. After field construction, the truck load with fully occupied sand was located on the right top of the pipeline. Measurements were performed three times at two month intervals to check the variation of the installed pipeline elevation.

**Table 8:** Cases for the second field test.

Type	Bedding and Foundation	Backfill
Case B1	Sand Bedding	180° and one Layer Sand + two Layer Original Soil
Case B2	50% Recycled Plastic + 50% New Plastic Foundation	Three Layers of *In-Situ* Soil

**Figure 16:**
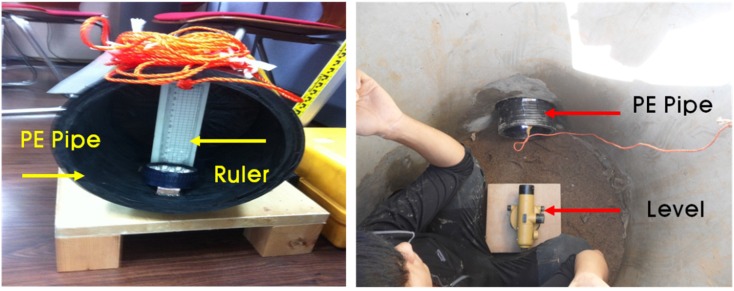
Measuring system of the second field construction.

To measure the pipeline elevation after field construction, a level and ruler (Figure 16) were used. First, the ruler was set at the beginning position inside of the PE pipe. The elevation of the pipeline was measured at 1 m intervals of 15 m pipeline using a level.

## 4. Conclusions

The research presented in this paper aimed to evaluate the performance of recycled plastic foundations for embedded pipelines. A small scale laboratory chamber test and two different field tests were conducted. Despite the possible limitations of the number of tests, the following conclusions can be drawn:

1) From the small scale laboratory chamber test, the applied loads at 2.5% and 5.0% of deformation were 3.45 kgf/cm^2^ and 5.85 kgf/cm^2^ for Case S1, and 4.42 kgf/cm^2^ and 6.43 kgf/cm^2^ for Case S2, respectively. These results means that the use of 50% recycled plastic foundation (Case S2) can endure larger loading than that of sand bedding (Case S1). For the case of lateral deformation, the measured deformations at 2.5% and 5% were 6.60 mm and 12.51 mm for Case S1, and 5.90 mm and 11.30 mm for Case S2, respectively. Therefore, Case S2 showed better resistance than Case S1.
2) The calculated flatness of Case S2 is lower than that of Case S1, which means that the PE pipe of Case S2 is more resistant than that of Case S1. The flatness of Case S1 and Case S2 at 6.0 kgf/cm^2^ of applied stress was 0.896 and 0.921, respectively.
3) From the first field test, the vertical deformation of the recycled plastic foundation (Case A2) was very small. According to the analysis based on the PE pipe deformation at the connection (CN) and at the center (CT), the pipe deformation at each part for Case A1 was larger than for Case A2, which adopted the recycled lightweight plastic foundation.
4) In general, as the load increased, the measured strain increased for all of the cases. In Case A1, the strain at S3 was smaller than at S1. In this case, the measured strains were not uniform at each measured location (S1, S2 and S3), which means that the applied overburden pressure, including the backfill and the backhoe load, was not uniform on the PE pipe due to the non-uniform compaction of sand bedding and backfill around the PE pipe. This non-uniform compaction induced the differential settlement. In Case A2, the measured strains at S1, S2 and S3 were relatively uniform. The use of a recycled lightweight plastic foundation uniformly supported the installed PE pipe, meaning that the overburden pressure was relatively uniformly distributed on the PE pipe.
5) From the second field test, the settlement of Case B2 with the recycled plastic foundation was smaller than that of Case B1 with sand bedding. The measured maximum settlements of Case B1 and Case B2 were 1.05 cm and 0.54 cm, respectively. The use of a plastic foundation can reduce the settlement of the embedded pipeline.
